# Refreshing Multi-Feature Objects in Visual Working Memory

**DOI:** 10.5334/joc.464

**Published:** 2025-09-30

**Authors:** Alessandra S. Souza

**Affiliations:** 1 Center for Psychology, Faculty of Psychology and Education Sciences, University of Porto, Portugal

**Keywords:** binding, retro-cue, attention, colored shapes, working memory unit

## Abstract

“Thinking of” a representation in working memory is assumed to refresh its trace, boosting its accessibility. We previously demonstrated that think-of cues can be used to guide the refreshing of individual features in working memory (e.g., colors, orientations, words), with items refreshed more often being better reproduced in recall tasks. In the present study, we tested whether refreshing modulates the accessibility of multi-feature objects, contributing either to the maintenance of feature bindings or individual features. The “think-of” cues procedure was combined with a recognition task in Experiments 1 (N = 31) and 2 (N = 77) and with a dual-feature report task in Experiment 3 (N = 117). In all studies, participants encoded four colored shapes. During retention, a sequence of three think-of cues was presented, guiding refreshing of the memoranda 0, 1, or 2 times. In Experiments 1 and 2, a colored shape was presented for recognition (50% match and 50% mismatch). Critically, mismatch probes consisted of intrusions (new color with old shape or old color with new shape). Refreshing monotonically improved match-probe recognition, but not the rejection of intrusion probes. In Experiment 3, refreshing increased the correct recall of both features of the same object, whereas the probability of a single correct report remained constant. These results suggest that refreshing acted on the representation of the integrated object. Not refreshed objects, however, did not become more fragile to binding disruption, they mostly lost accessibility in an all-or-none fashion.

Most of the objects we encounter in our daily lives are composed of multiple features. When we see an object in front of us – let’s say an apple in a fruit basket, we become aware of its multiple visual features, such as its color, shape, orientation, size and texture, among others. Although different neurons in our brains are sensitive to each of these properties (Livingstone & Hubel, 1988), our visual experience is of an integrated, bound object – a big, red apple. So far, it is still unclear how visual features become bound during visual perception, and how this integrated representation is maintained in memory (Bays et al., 2024; Schneegans & Bays, 2019). In the present study, we are concerned with the role of attention in the maintenance of an integrated object in visual working memory. More specifically, we examined if attention modulates the maintenance of the bindings between the multiple features of an object.

## The Role of Attention in Feature Binding

There are two opposing views regarding the role of attention for feature binding. On the one hand, the *attention-demanding binding hypothesis* originates from the view that attention contributes to feature binding during perception (Treisman & Gelade, 1980); accordingly, it assumes that the storage of the bound object in memory continues to demand attention (Kahneman et al., 1992). It predicts that the disruption of attention leads to the disintegration of the bindings, leaving only memory for the individual features of the object. On the other hand, the *automatic binding hypothesis* proposes that the retention of bindings in memory is as demanding as the retention of simple individual features, occurring largely automatically after the representation is formed (Luck & Vogel, 1997; Vogel et al., 2001). According to the latter view, if attention contributes to the maintenance of representations in working memory, its role would be similarly important for individual features as for feature bindings.

## Evidence From Dual-Task Paradigms

The testbed for these hypotheses has been the dual-task paradigm. The logic of this paradigm is that imposing an attention-demanding distraction task during the retention interval of a memory task will prevent the deployment of attention to memory representations. By varying the requirements to memorize individual features (e.g., only colors or only shapes) vs. feature bindings (e.g., colored shapes), one can assess which type of representation is more sensitive to dual-task costs. If binding maintenance demands attention, dual-task costs should be larger for storing bindings compared to individual features. Conversely, if bindings are maintained automatically, dual-task costs should be of similar magnitude for features and bindings.

The dual-task approach has produced mixed results. Some studies observed larger dual-task costs for the retention of bindings – for example, the exact combination of color and shape of an object – compared to individual features, i.e., only the color or only the shape of the object (Brown & Brockmole, 2010; Elsley & Parmentier, 2009; Fougnie & Marois, 2009; Gao et al., 2017; He et al., 2020; Lu et al., 2019; Peterson & Naveh-Benjamin, 2017; Shen et al., 2015; Zokaei et al., 2014). Conversely, other studies observed similar dual-task costs for features and bindings (Allen et al., 2006, 2012; Goldenhaus-Manning et al., 2024; J. S. Johnson et al., 2008; C. C. Morey & Bieler, 2013; Vergauwe et al., 2014). It is clear, therefore, that we need additional sources of evidence to settle on the role of attention for feature bindings. The present research focuses on a more direct approach to assessing how attention contributes to feature bindings by manipulating how much attention is deployed to each memory representation during the retention interval.

## Guiding Attention to Memory Representations

Focusing attention briefly on each memory representation is known as *attentional refreshing* (for a review see Camos et al., 2018). Attentional refreshing is assumed to strengthen the refreshed representation in working memory making it more robust to forgetting (Barrouillet et al., 2011; M. K. Johnson, 1992). In our previous work, we refined a procedure to guide the attentional refreshing of individual features in working memory (Atkinson et al., 2022; Loaiza & Souza, 2022; Souza et al., 2015, 2018; Souza & Oberauer, 2017; Vergauwe et al., 2023). In our typical paradigm, participants memorized an array of colored dots for a continuous reproduction test using a color wheel. With this test procedure, we can measure how precisely information is retained in memory by examining the error with which the tested feature is reproduced. To manipulate attention deployment, a sequence of four retro-cues (central arrows) was presented during the retention interval (Griffin & Nobre, 2003; Landman et al., 2003; for a review of the retro-cue procedure see Souza & Oberauer, 2016). Each cue pointed to the location where a memory object previously appeared. Participants were told that their main task was to *think of* the object that was indicated by the retro-cue. Across the sequence of four cues, the memory objects could be refreshed 0 (never cued), 1 (cued once), or 2 times (cued twice). Unlike most studies using retro-cues, these “think-of” cues were only modestly predictive (33% at best) of the to-be-tested object, and hence they could not be used to prioritize a single representation for the test or to remove uncued ones. The goal was to motivate participants to simply direct their attention to the cued representation, thereby refreshing it. At the end of the retention, one memory object was cued to be recalled. Memory objects refreshed 0, 1, and 2 times were tested in an equal number of trials. Overall, these studies converged in showing a decrease in the recall error as a monotonic function of the number of times the memory representation was refreshed. These results were observed for the recall of continuous colors (Loaiza & Souza, 2022; Souza et al., 2015, 2018; Souza & Oberauer, 2017), continuous orientations and, to a smaller extent, words (Souza et al., 2018). In sum, they indicate that the amount of attention devoted to simple features during the maintenance phase modulates how well these representations can be retrieved from working memory. So far, it is unclear if refreshing modulates the retention of bindings or if it only strengthens individual features in working memory.

## The Role of Refreshing for Bindings

One prior study presented evidence consistent with attentional refreshing strengthening the binding of an object to its spatial location (aka context-feature binding). Rerko and Oberauer (2013) asked participants to memorize an array of six colored dots arranged around an invisible circle. During the retention interval, a series of 1–3 retro-cues were presented pointing to the locations previously occupied by the memory objects. At the memory test, a central probe appeared, and participants had to indicate if it matched the color of the last cued object. The probe matched the last cued object in 50% of the trials and mismatched it in the remaining trials. Mismatch probes could be new colors (colors not presented in the memory array; aka negative probes) or the color of a memory object that was not the last cued one, aka an intrusion probe. Due to their higher familiarity, intrusion probes are harder to reject than negative probes – producing lower accuracy and slower reaction times (RTs). Rerko and Oberauer focused on intrusion costs to assess whether cueing strengthened the cued feature or the binding of the feature to its location. In their study, intrusion probes could be objects that were not cued or that were cued previously in the sequence. Given that the number of cues was unpredictable, participants had to focus on the cued object with each presented cue and shift their focus of attention if a new cue appeared. Accordingly, Rerko and Oberauer reasoned that if the cue directs attention to the memory representation and its feature becomes strengthened – for example, participants were cued to the location where a yellow dot appeared and yellow increased in strength in memory – then it would be harder to reject this intrusion color compared to other not cued colors that were also stored in memory. If refreshing strengthens the binding, however, participants would be better able to reject a previously cued color because they know this color was not at the last cued location – this would mean that in being cued to focus attention at the yellow that appeared at the top, the association of yellow to that specific location was strengthened and not simply the representation of yellow. They indeed observed that previously cued intrusions were easier to reject than non-cued intrusions, in line with refreshing strengthening feature-location bindings. Location-feature bindings are, however, one special case of binding, which involves the spatial context in which the item appeared, which is extrinsic to the object. Other features such as the color and the shape of the object, are considered intrinsic features because they define the object itself (Ecker et al., 2013; Goldenhaus-Manning et al., 2024). Here, we wanted to consider especially the binding of intrinsic features of the object.

Gajewski and Brockmole (2006) investigated the role of attention to the maintenance of multi-feature objects in working memory. They used an unpredictive exogenous retro-cue (a flash presented at the location previously occupied by a memory object) to guide attention to the representation of one colored shape during the retention interval. At the memory test, they randomly probed one memory location, and participants had to verbally recall both features of the probed object. The reasoned that the cue would draw attention to the cued object, thereby pulling attention away from the uncued objects. Accordingly, if attention was required to maintain feature bindings, they expected that the correct recall of the feature conjunction of uncued objects would suffer, with a corresponding increase in the recall of individual features (i.e., features would be recalled in the wrong combinations). They observed that recall of the conjunction decreased in the invalid condition compared to the valid condition, but this was not accompanied by an increase in the recall of individual features, but rather by a failure to recall either feature correctly. They concluded that attention was not necessary for the maintenance of an integrated object – feature-bindings did not fall apart without attention, but rather attention helps retention of the integrated object that fails in an all-or-none fashion.

In the present study, the aim was to build upon these studies to provide more evidence for the role of attention in maintaining integrated objects.

## The Present Study

**Figure 1:**
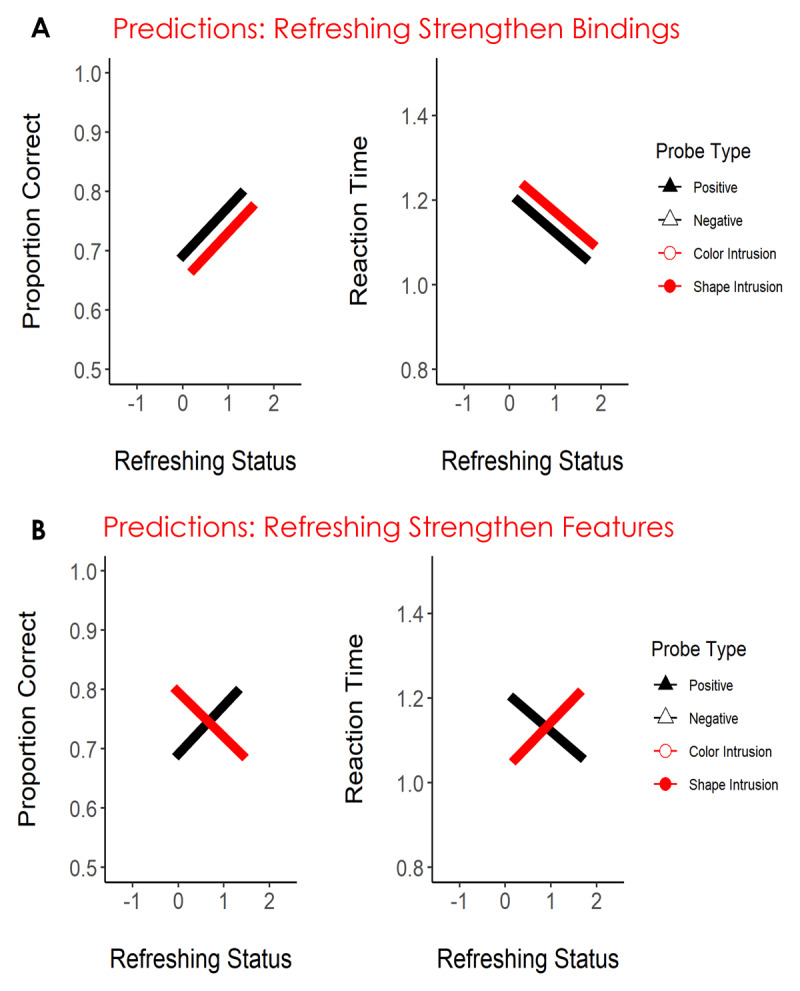
Predictions of Strengthening of Bindings **(Panel A)** and Strengthening of Individual Features **(Panel B)** for Accuracy and Reaction Time in Experiments 1 and 2. *Note*. The black line represents positive probes and the red line the intrusion probes. We had no expectation of a difference between color and shape intrusions, hence we depicted intrusion probes with a single line. Negative probes are uninformative for distinguishing these hypotheses and hence they were omitted from the illustration.

In the present study, we took leverage of the think-of cues procedure to guide attention to multi-feature objects in working memory. In Experiments 1 and 2, we combined the think-of-cues procedure with a recognition task inspired by the study of Rerko and Oberauer (2013). Participants encoded a sequence of four colored shapes. During the retention interval, three think-of cues were presented guiding attention to a subset of the memory objects. We used this procedure to modulate the refreshing status (0, 1, or 2 times) of the memory objects. Then, a central probe was presented, and participants had to judge if the probe matched or mismatched one of the memory objects (i.e., the exact color-shape combination). The probe matched one memory object in 50% of the trials and mismatched it in the remaining 50%. As in Rerko and Oberauer (2013), mismatch probes could be negative (i.e., both the color and the shape were new) or intrusions (either the color or the shape was presented in the memory array, but they were recombined with a new feature). Our aim was to use performance on match and intrusion probes to distinguish between the role of attention in strengthening bindings vs. features. Figure 1 illustrates our predictions. First, we expected to observe a refreshing frequency effect for accepting match probes, such that accuracy would be higher and reaction times faster, the higher the refreshing status of the object. This would count as a direct replication of the refreshing frequency effect we previously observed, now using a recognition paradigm with multi-feature objects. That being the case, we reasoned that performance for intrusion probes would be informative regarding whether bindings or features were strengthened. If refreshing strengthens bindings, then we thought participants would find it easier to reject intrusion probes the more the feature that appeared in the probe had been refreshed because they know this was not the correct feature combination. Conversely, if refreshing strengthens features, the opposite would be the case: it would be harder to reject an intrusion probe that contained a refreshed feature.

In Experiment 3, we combined the think-of-cue procedure with a dual-report task (Bays et al., 2009; Fougnie et al., 2013; Fougnie & Alvarez, 2011; Gajewski & Brockmole, 2006; Hadhrami et al., 2023; Schneegans & Bays, 2017), following more the logic of Gajewski and Brockmole (2006). Participants were tasked with remembering the color, shape, and location of each object. At test, one feature was shown on the screen center (e.g., a color), thus serving as the recall cue to report the remaining two features (shape and location) of the memory object. Here, we assessed the probabilities of getting one response correct vs. both responses correct in the dual test as a function of refreshing status. If refreshing strengthens bindings, we should observe a selective increase in the probability of getting both responses correct as a function of refreshing.

## Experiment 1

### Method

#### Openness and Transparency

All the stimuli and task materials, together with the anonymized data and analysis scripts reported here, are available at the Open Science Framework at https://osf.io/u9368/ (Souza, 2025). The studies were not preregistered.

#### Participants

A total of 31 students from the Psychology Department of the University of Zurich (4 men, 27 women; mean age = 24 years, age range 18–35) participated in two 1-hour laboratory sessions in exchange for extra-course credit or monetary reimbursement (15 CHF per hour). Participants were tested individually in private booths. Participation criteria include being between 18–35 years old, with normal color vision and acuity, and German knowledge sufficient to understand the instructions.

All participants completed an informed consent form before starting the experiment and were debriefed at the end. The experimental protocol was deemed safe as assessed by the ethical self-assessment checklist implemented by the Institutional Review Board at the University of Zurich.

#### Stimuli and Procedure

**Figure 2:**
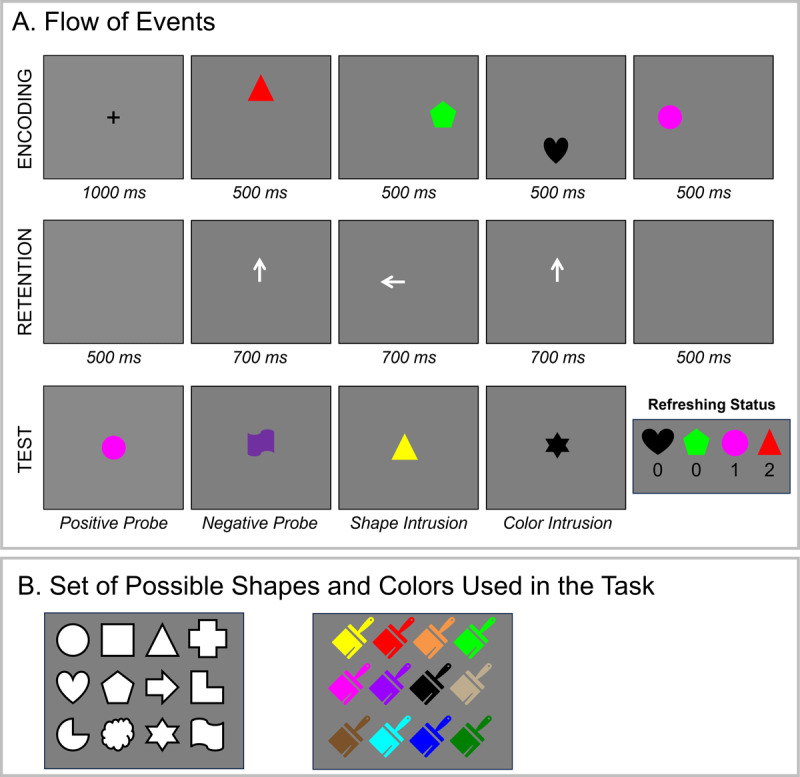
Illustration of the Experimental Procedure **(Panel A)** and Set of Possible Stimuli **(Panel B)** in Experiment 1.

Experiment 1 was programmed using the Psychophysics Toolbox (Brainard, 1997; Pelli, 1997) implemented in Matlab. The stimuli consisted of colored shapes. We used sets of 12 easily distinguishable shapes and colors (see Figure 2B) in Experiment 1, totaling 144 possible combinations.

Figure 2A illustrates the procedure in Experiment 1. Each trial started with the presentation of a white fixation cross (duration = 1000 ms) against a grey background. Thereafter, a sequence of four colored shapes (radius = 50 pixels) appeared one by one across four positions (180 pixels away from the screen center): top, right, bottom, left – always in that order (duration = 500 ms). Colors and shapes were never repeated within a trial. After a 500-ms retention interval, a sequence of three central retro-cues (duration = 700 ms) was presented, followed by another 500-ms post-cue interval. Participants were instructed that they should think of the memory object indicated by the cue for as long as the cue was onscreen. The three cues could point to three different memory objects (e.g., top-left-right), or they could point to one object twice (e.g., top-left-top) and another once. Two successive cues could not point to the same object. This manipulation allowed us to create memory objects with three levels of refreshing status: 0, 1 or 2, depending on the frequency with which the objects were cued to be refreshed. Features of objects refreshed 0, 1, and 2 times were equally likely to be tested at the end of the trial.

At the end of the trial, a memory probe appeared in the center of the screen, and participants had to judge whether the probe matched one of the memory objects (i.e., same color-shape combination) by pressing the left (match) or right (mismatch) arrow key on the keyboard. In 50% of the trials, the probe was a match (aka positive probe). In 10% of the trials, the probe presented a color and shape that was not part of the memory array (aka negative probe). In 20% of the trials, the probe contained a shape that was part of the memory array in a non-presented color (aka shape intrusion), and in the remaining 20% of the trials, the probe presented a color that was part of the memory list combined with a new shape (color intrusion). The probe never contained two recombined features from different objects. The probe remained onscreen until a decision was made. Participants were instructed to respond as fast and as accurately as possible. Immediate feedback (the words correct or incorrect) was presented for 500 ms before the start of the next trial. Each session consisted of 10 practice trials and 240 trials, hence there were 480 trials for analysis in total for each participant. Participants completed the task under articulatory suppression (repetition of the syllable “bababa” throughout the study phase).

### Data Analysis

The dependent variables of interest in the current study were the proportion of correct responses and the reaction time (RT) for each probe-type as a function of the refreshing status (0, 1, or 2) of the features. Experiment 1 also included negative probes which had no refreshing status (hereafter considered as –1).

We selected for analysis only RTs of correct responses. This led to the retention of 69% of the responses. Outlier RTs were further trimmed by applying the median absolute deviation procedure proposed by Leys et al (2013) with a criterion of 3, which was deemed very conservative. We applied this procedure for each participant in each experimental condition. This procedure led to the removal of an additional 5.38% of the trials.

We analyzed the data using BayesFactor package (R. D. Morey & Rouder, 2015) implemented in R (R core team, 2017). We used the default settings of the package to run one-way Bayesian ANOVAs and t-tests. The default settings include a Cauchy prior distribution with scale r ≈ 0.707 on the standardized mean difference (effect size) for both ANOVAs and t-tests. Bayesian analysis provides a Bayes Factor (BF) which reflects the relative evidence for two models: the alternative model (M1) that includes the effect under evaluation, and a Null model (M0) that does not include the effect. We will report BF_10_, which represents the evidence in favor of M1 over M0. The evidence for M0 over M1 can be computed as 1/BF_10_. A BF_10_ between 3 and 10 is considered moderate evidence for an effect, and values above 10, strong evidence. A BF_10_ between 0.33 and 0.1 indicates moderate evidence for the Null hypothesis, whereas values below 0.1 indicate strong evidence for the Null (Jeffreys, 1961; Kass & Raftery, 1995).

### Results

**Figure 3:**
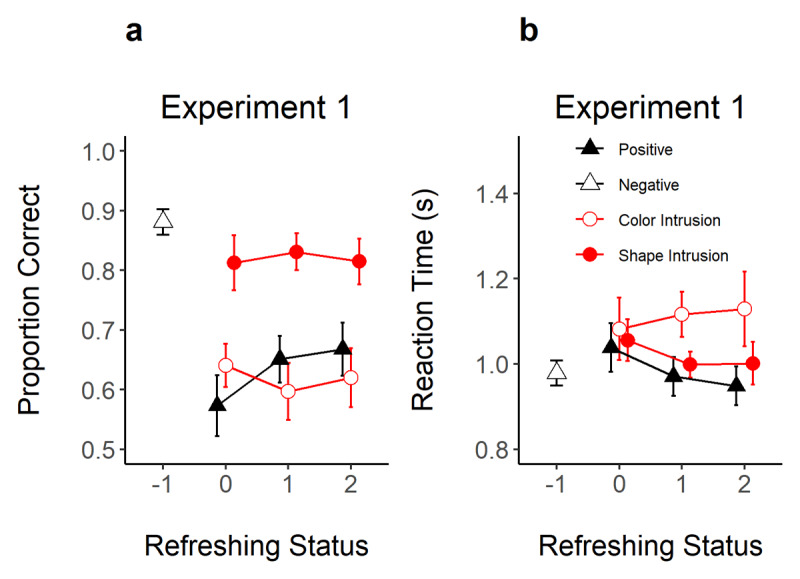
Proportion of Correct Responses **(Panel A)** and Reaction Time **(Panel B)** in Experiment 1 as a Function of Refreshing Status (x-axis) and Probe Type (Different Shapes). *Note*. Error bars represent 95% within-subjects confidence intervals.

Figure 3 shows the proportion of correct responses and RT as a function of refreshing status for each probe type. We assessed separately the evidence for an effect of refreshing status on the acceptance of positive probes, and on the rejection of color and shape intrusion probes. Table 1 presents the evidence for an effect of refreshing status on these dependent variables, and for pairwise contrasts between refreshing levels within positive probes.

**Table 1:** Evidence (BF_10_) for the Effect of Refreshing Status on the Proportion of Correct Responses and RTs for Each Probe Type in Experiments 1 and 2, and for the Pairwise Contrast of Refreshing Levels Within Positive Probes.


	PROPORTION CORRECT		RTs	
	
	E1	E2	E1	E2

**Probe Type**				

*Positive*	76.5 (±0.63%)	12949 (±0.93%)	3.36 (±0.51%)	20.10 (±0.33%)

*Color Intrusion*	0.32 (±0.72%)	0.05 (±0.77%)	0.14 (±0.70%)	0.05 (±0.22%)

*Shape Intrusion*	0.15 (±0.67%)	0.06 (±0.92%)	0.64 (±0.63%)	0.15 (±0.35%)

**Contrast of Refreshing Levels within Positive Probes**				

*0 vs. 1*	5.89	31.18	1.08	0.19

*0 vs. 2*	22.13	3125	6.12	12.25

*1 vs. 2*	0.32	1.28	0.27	7.06


#### Positive Probes

As shown in Figure 3, refreshing increased the correct recognition of positive probes and reduced RTs. As shown in Table 1, the evidence for an effect of refreshing frequency on proportion correct was strong (BF_10_ = 76.5), but only worth mentioning in RTs (BF_10_ = 3.36). We also conducted t-tests to assess the evidence for differences between refreshing levels. Evidence was substantial for a difference between not-refreshed (0 refreshings) compared to refreshed objects (1 and 2 refreshings; BF_10_ = 5.89 and 22.13) in proportion correct. However, there was ambiguous evidence for a difference between objects refreshed once vs. twice (BF_10_ = 0.32). In RT, there was only substantial evidence for faster RTs when moving from 0 to 2 refreshing steps (BF_10_ = 6.12).

#### Intrusion Probes

Although refreshing frequency improved the recognition of positive probes, the correct rejection of color and shape intrusion probes was not affected by the refreshing status of the intrusion feature (BF_10_ = 0.32 and 0.15). Likewise, RTs to reject these probes did not show evidence for an effect of refreshing status (BF_10_ = 0.14 and 0.64).

#### Intrusion Costs

The lack of an effect of refreshing on intrusion probes was not due to intrusion probes not exerting an effect on performance. Experiment 1 also included negative probes, thereby allowing us to gauge the evidence for intrusion costs. Accuracy was higher for negative probes than for intrusion probes containing a memory shape (BF_10_ = 138.7) or color (BF_10_ = 4.87 × 10^9^). RTs were also faster for negative probes compared to color intrusion probes (BF_10_ = 939.2), but there was ambiguous evidence for a difference to shape intrusions (BF_10_ = 1.29). Hence, presenting only one of the features of a memory object incurred in decision costs. Critically for our research question, however, these decision costs were not modulated by refreshing frequency.

### Discussion

Experiment 1 extended the refreshing frequency effect to a recognition task with multi-feature objects. Although we expected that refreshing would affect both the probability of accepting positive probes and intrusion probes, Experiment 1 only showed evidence for a refreshing frequency effect for positive probes. This indicates that attentional refreshing of multi-feature objects improves the recognition of probes that match the memory item in both relevant features. We reasoned that if individual features were strengthened by attentional refreshing, then it would become harder to reject a probe that contained a refreshed feature (color or shape intrusions). Conversely, if attentional refreshing strengthened feature bindings, then it would be easier to reject intrusion probes because it would become increasingly apparent that the presented feature was in the wrong combination. Neither of these predictions panned out.

Since this was the first time we employed a recognition task to measure the impact of refreshing multi-feature objects, we decided to replicate the study with a modified procedure that took care of a few suboptimal design characteristics. First, in Experiment 1, participants were cued to a spatial location with an arrow, but there might have been imprecision regarding the encoding of location information since location was irrelevant to the task. Additionally, central cues require more effort to process than peripheral cues and take longer to induce a cueing benefit (Han et al., 2023). Therefore, we reasoned that marking the spatial locations with placeholders and using peripheral cues could facilitate refreshing. Second, Experiment 1 included 700 ms per refreshing cue, whereas most previous studies presented cues for 500 ms. Loaiza and Souza (2022) and Vergauwe et al. (2024) observed that using longer cue times (1000 ms) hindered performance compared to the usual 500 ms duration. Accordingly, we decided to reduce cue time to 500 ms. Third, there were some shapes and colors in Experiment 1 that were less familiar (e.g., incomplete circle and square; beige color), which we decided to remove from the next experiment. Fourth, Experiment 1 involved data collection in the laboratory, which is more labor-intensive. Since online experiments allow for data collection of larger samples, we decided to move the study to an online platform to increase the number of participants, potentially allowing us to have more decisive evidence for or against the presence of effects.

## Experiment 2

As in Experiment 1, Experiment 2 involved a recognition task with multi-feature objects. Participants were presented with a sequence of four colored shapes and then cued to refresh some of the shapes. We marked the spatial locations of the objects with placeholders and used peripheral retro-cues to reduce spatial uncertainty and to more effectively guide attentional refreshing. We also reduced the duration of the refreshing cues to 500 ms. We expected that these changes could increase the refreshing frequency effect, thereby potentially allowing us to more credibly detect changes in the response to intrusion probes. Experiment 2 also involved some other minor changes in the layout of the task (white background, layout of the placeholders), which were related to the fact that code for online data collection of a color-shape task was already available and had been successfully piloted before. We did not expect these changes to be of relevance to the effects of interest in the current study, and hence we considered that results should generalize despite these trivial changes. Data collection was also online which allowed for recruitment of a large number of participants, and hence more credible estimation of effects.

### Participants

Students from the Psychology Department of the University of Porto were invited to take part in two 30-min online sessions in exchange for extra-course credit. Participants enrolled by signing up on an online platform, and the study was available for an entire semester. We accepted responses from interested participants. After enrolling, participants received the link to the first session per email. After completing the first session, they would send an email to the experimenter to receive the link to the second session. Sixty-eight participants completed the two sessions. Additionally, six participants completed only one experimental session (not responding to the second invitation email), and three completed three sessions (they visited one of the links twice). Given that memory trials were randomly created in every visit to the study, we included all available data of all participants (N = 77; 9 men, 67 women, 1 not say; mean age = 20.9 years, age range 18–48). Participation criteria include being older than 18 years old and having normal color vision and acuity. The study was available both with Portuguese and English instructions (for Erasmus students).

All participants completed an online informed consent form before starting the experiment and were debriefed at the end. The institutional review board of the Faculty of Psychology and Education Sciences of the University of Porto approved the experimental protocol used in Experiments 2 and 3 (approval 2023/08-05).

### Stimuli and Procedure

**Figure 4:**
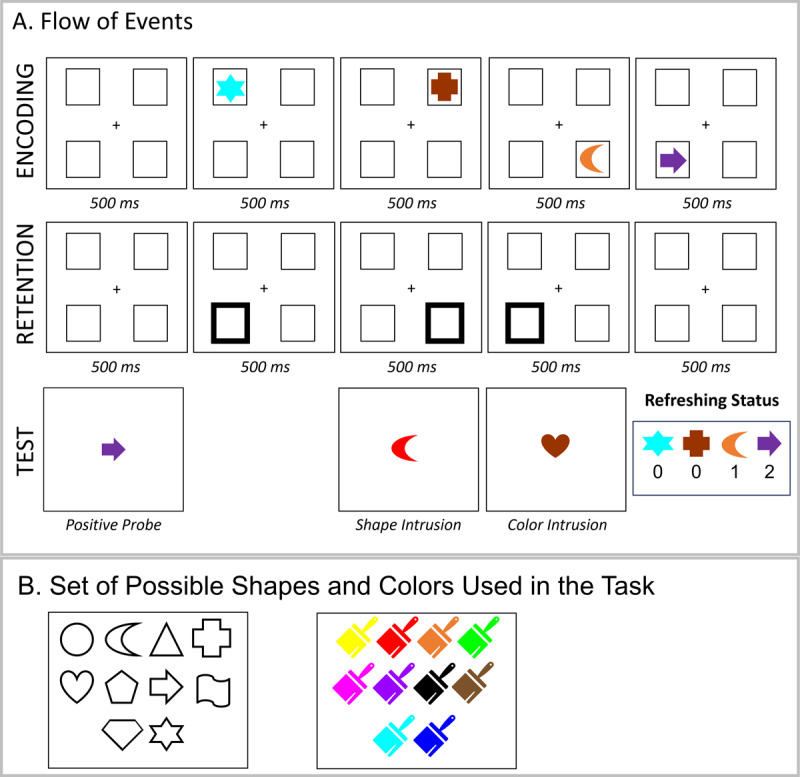
Illustration of the Experimental Procedure **(Panel A)** and Set of Possible Stimuli **(Panel B)** in Experiment 2.

Experiments 2 and 3 were programmed using the open-source lab.js online experiment builder (Henninger et al., 2022). Experiments 2 and 3 were deployed online using the freely available server MindProbe (https://mindprobe.eu/) sponsored by ESCOP and Journal of Cognition. We used a reduced set of 10 shapes and 10 colors in Experiments 2 and 3 (100 possible combinations). Figure 4B presents the set of possible shapes and colors.

Experiment 2 consisted of a conceptual replication of Experiment 1 using a slightly different task setup. Each experimental trial started with the presentation of four placeholders (see Figure 4A) against a white background for 500 ms. Thereafter, the sequence of four memory objects was presented (each for 500 ms). This was followed by a 500-ms retention interval, and then a sequence of three peripheral retro-cues (i.e., the highlight of one of the placeholders where a memory object appeared), and a final 500-ms blank interval. The duration of each cue was 500 ms. We used peripheral retro-cues to more efficiently guide attention to the cued location (Han et al., 2023), and we reduced cue time as our previous studies showed that 500 ms was sufficient for producing a refreshing effect and led to better performance than longer cue presentation (Loaiza & Souza, 2022).

As in Experiment 1, memory objects were cued to be refreshed 0, 1, or 2 times. Participants were instructed to think of the object that appeared in the highlighted location for as long as the cue was onscreen. At the end of the trial, a central memory probe was shown, and participants indicated whether the color-shape combination was part of the memory list by pressing the A or L keys in the keyboard for a match-mismatch response, respectively. Experiment 2 did not contain negative probes. In 50% of the trials, a match probe was presented. In the remaining 50%, either a shape (25%) or color (25%) intrusion probe was presented. Participants received feedback regarding the correctness of their response.

Participants completed 4 practice trials and 200 test trials per session, totaling 400 trials for analysis. No articulatory suppression was requested, as previous studies have not found a difference in the refreshing effect with or without the use of articulatory suppression (Souza et al., 2015), and it was not possible to monitor compliance with this procedure in an online setting.

### Data Analysis

The dependent variables of interest in the current study were the proportion of correct responses and RTs for each probe type as a function of the refreshing status (0, 1, or 2) of the features. We selected for analysis only RTs of correct responses, which led to the retention of 65.43% in Experiment 2. The same outlier procedure as in Experiment 1 was applied to the RTs, implying the exclusion of 5.69% of the trials. All remaining analysis details were as in Experiment 1.

#### Positive Probes

**Figure 5:**
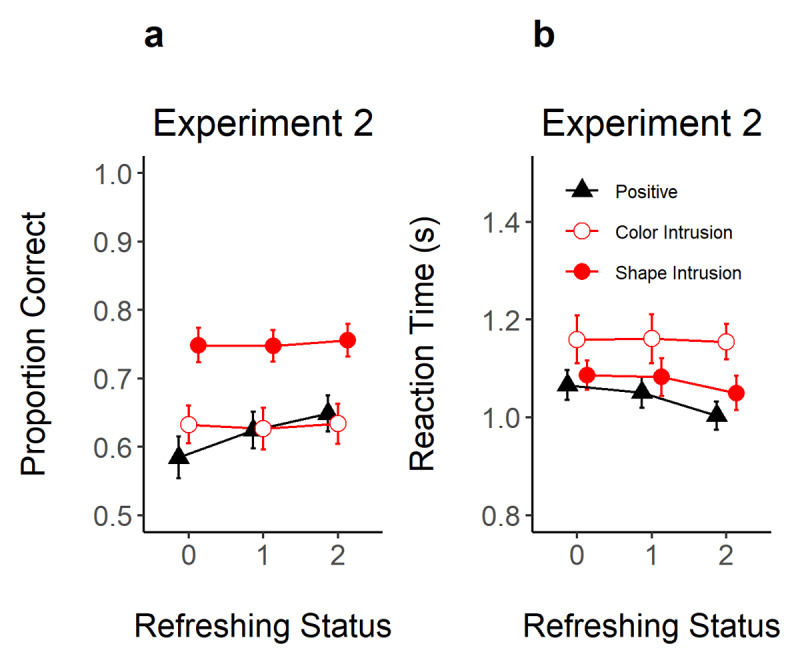
Proportion of Correct Responses **(Panel A)** and Reaction Time **(Panel B)** in Experiment 2 as a Function of Refreshing Status (x-axis) and Probe Type (Different Shapes). *Note*. Error-bars represent 95% within-subject confidence intervals.

As shown in Figure 5, refreshing increased the correct recognition of positive probes and reduced RTs in Experiment 2. As shown in Table 1, the evidence for an effect of refreshing frequency was very strong (BF_10_ = 12949) and strong (BF_10_ = 20.1) in proportion correct and RTs. We also conducted t-tests to assess the evidence for differences between refreshing levels. In proportion correct, there was strong evidence for a difference between not-refreshed (0 refreshing) compared to refreshed objects (1 and 2 refreshing steps, BF_10_ = 31.18 and 3125, respectively). However, there was ambiguous evidence for a difference between objects refreshed once vs. twice (BF_10_ = 0.27). In RT, there was substantial evidence for faster RTs when moving from 0 to 2 refreshing steps (BF_10_ = 12.25) and 1 to 2 refreshing steps (BF_10_ = 7.06).

#### Intrusion Probes

Although refreshing frequency improved the recognition of positive probes, the correct rejection of color and shape intrusion probes was not affected by the refreshing status of the intrusion feature (BF_10_ = 0.05 and 0.06 for color and shape, respectively). Likewise, RTs did not show evidence for an effect of refreshing status, with weak to moderate against an effect of refreshing status for color (BF_10_ = 0.05) and shape (BF_10_ = 0.15) intrusion probes.

### Discussion

Experiment 2 replicated and extended on the paradigm used in Experiment 1. We improved the task setup to facilitate the observation of refreshing frequency effects, and we also doubled the sample size. These modifications were successful in producing more credible evidence for and against refreshing frequency effects (see Table 1 for the evidence in each of the Experiments). Nevertheless, the pattern of findings was similar across the two studies: refreshing frequency modulated only the acceptance of positive probes. These results confirm that the pattern of findings is robust and generalizes across different task setups (changes in cueing mode, task layout, data-collection setting, use or not of articulatory suppression, sample/culture, etc).

One limitation of the present study was that we avoided recombining features from two memory items – we always presented only one single memory feature combined with a new feature. This choice was made to better control the impact of each feature, given that it might be difficult to predict how the recombination of features with different refreshing levels would affect performance. Another limitation was that we made no explicit mention that each feature would only be used once in each trial. However, to reject intrusion probes (e.g., a red circle) based on the knowledge that we have in working memory another object with one of these features (e.g., we remember a red triangle), one might need to develop such a rule. Future studies could try to instruct this rule explicitly to see if rejection of intrusion probes improves with higher refreshing frequency.

Perhaps the use of a recognition task was not best suited to addressing the question of whether refreshing strengthens individual features or feature bindings. Recent studies have focused on dual-report paradigms in which participants are cued to report the multiple features of a multi-feature object (Bays et al., 2009, 2011; Fougnie et al., 2013; Fougnie & Alvarez, 2011). At the memory test, participants receive one feature as a retrieval cue and reproduce the remaining two features. This type of test allows for assessing the stochastic dependency between the two responses, which reflects the accurate retrieval of the binding. Accordingly, Experiment 3 implemented a dual-report paradigm.

## Experiment 3

Experiments 1 and 2 used a recognition task to assess the role of refreshing for the maintenance of multi-feature objects. In Experiment 3, we implemented a dual-report task in which participants were asked to retain the color, shape, and location of each memory object to reproduce two of these features when prompted with the third one. This allowed us to assess the role of refreshing for the retrieval of each feature separately and the likelihood of retrieving both features of the same object. If refreshing strengthens bindings, then it should selectively affect the probability of recalling correctly both features of the same object.

### Methods

#### Participants

Students from the Psychology Department of the University of Porto were invited to take part in two 30-min online sessions in exchange for extra-course credit. All interested participants were allowed to take part. A total of 117 students took part in at least one session. Seven participants only completed one session, six participants completed three sessions, and 103 completed the two requested sessions. As in Experiment 2, we included the data of all participants in the analysis, given that trials were randomly generated for each run of the study. We only gathered demographic data of 115 participants who completed the first session (mean age = 21 years, age range 18–60; 96 women, 14 men, 1 rather not say, 3 non-binary, and 1 agender). Participation criteria include being older than 18 years old and having normal color vision and acuity. The study was available both with Portuguese and English instructions (for Erasmus students).

#### Stimuli and Procedure

**Figure 6:**
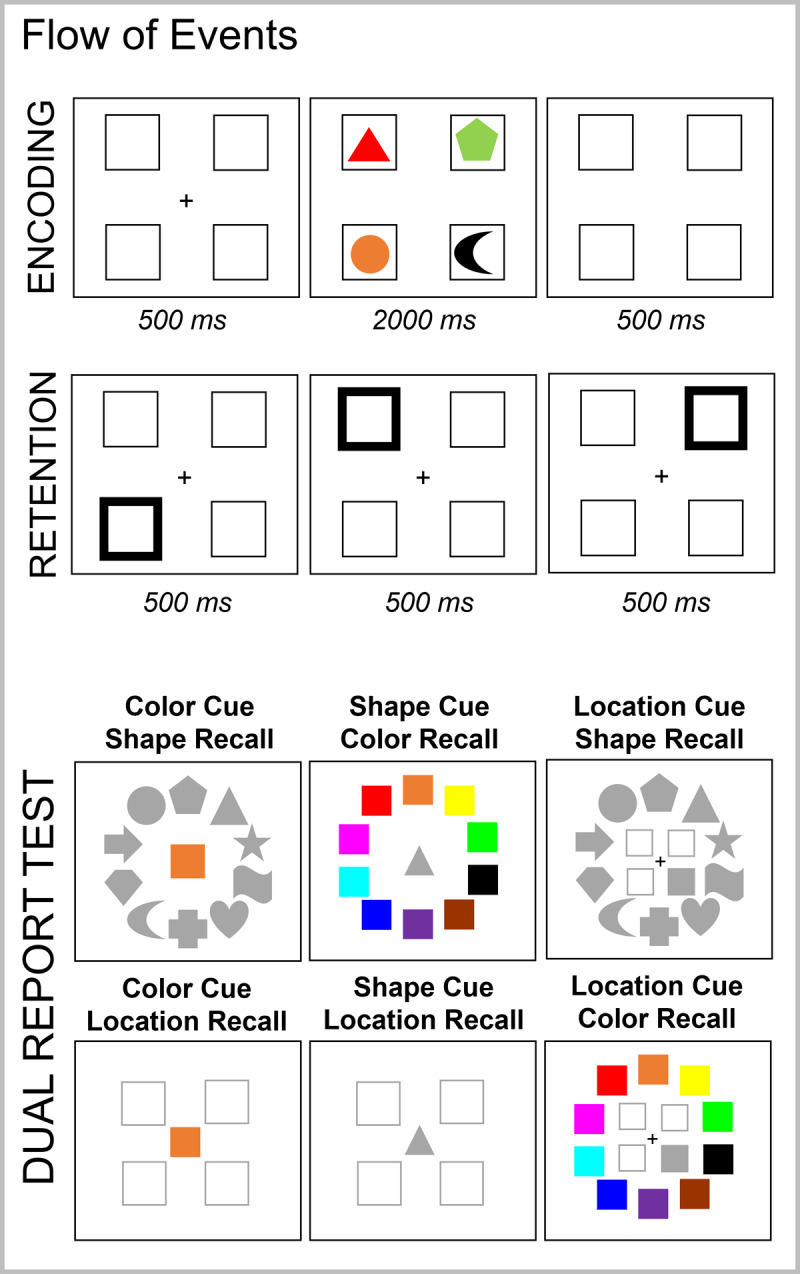
Illustration of the Flow of Events in Experiment 3.

Figure 6 illustrates the task procedure. We used the same materials and set-up as in Experiment 2, with the following three differences. First, to reduce the number of contextual cues that could be used to retrieve the memory objects, we presented all four stimuli simultaneously onscreen for 2000 ms. This removed the role of time as a potential retrieval cue. Second, we substituted the recognition test for a double-feature report test. Participants were presented with one feature of the target memory object and had to recall the other two features. As shown in Figure 6, when color was the retrieval cue, a colored square was presented at the center of the screen as a retrieval cue and participants had to retrieve the shape and the location in which that color was presented. In the shape recall test, the color cue was surrounded by all 10 possible shapes, whereas in the location recall test, it was surrounded by the four location placeholders. Participants had to click on the shape and location associated with that color. A similar procedure was implemented when shape or location served as retrieval cues as illustrated in Figure 6. The order of the two feature tests was randomly determined in each trial. Note that with this procedure, the color and shape recall tests allowed participants to respond with the correct target feature, with a non-target feature (the feature of one of the remaining memory items), or with an extra-list feature (a feature not presented in the current memory list). For location recall, however, participants could only report the target or a non-target location. We did not include extra locations because this could create uncertainty regarding the object cued to be refreshed, and hence dilute the refreshing frequency effect. Third, participants completed 4 practice trials and 176 test trials per session, totaling 352 trials for analysis.

As a reminder, the refreshing steps were implemented as in Experiment 2, namely a sequence of three peripheral cues was presented (each for 500 ms) and participants were instructed to think back to the object presented at that location. Before and after the start of the refreshing procedure, there was a blank interval for 500 ms, in which only the placeholders were visible.

#### Data Analysis

Given that this was a feature reconstruction task, we focused only on response accuracy. First, we evaluated the effect of refreshing status on the proportion of correct recalls of each feature. Second, we evaluated the effect of refreshing frequency on the correct recall of both features of the same object vs. the recall of a single feature. If refreshing strengthens the bindings, we expect an increase in the correct recall of both features as refreshing frequency increased.

### Results

**Figure 7:**
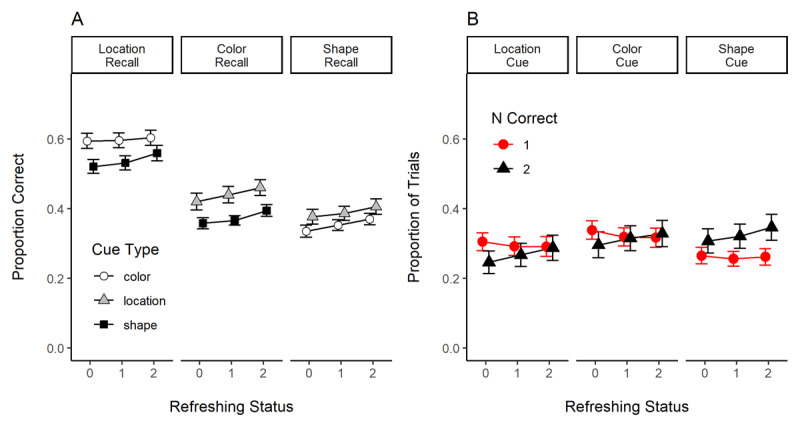
Results of Experiment 3. **Panel A**. Proportion of Correct Reports of Location, Color, and Shape as a Function of Refreshing Status and Recall Cue Type. **Panel B**. Proportion of Reports in a Trial with One Response Correct and With Both Responses Correct as a Function of Refreshing Status and Recall Cue Type.

Figure 7 presents the results of Experiment 3. Panel A shows the effect of refreshing status on the recall of each feature (location, color, and shape). When we examine performance as a function of the recalled feature, we observe a more substantial effect of refreshing for the recall of color and shape than of location. Table 2 shows, for each feature separately, the evidence for the effect of refreshing status, cue type, and their interaction. This table shows that refreshing frequency improved the recall of color and shape, but not of location, which was also the feature with better performance. This is arguably the case because the guessing rate for location is higher (25%) compared to the color and shape features (10%). We also explored whether refreshing selectively impacted the probability of recalling the feature of another object (lure) vs. an extra feature (not presented in the current trial) for the recall of color and shape. We did not uncover a credible pattern (data not shown). Overall, this analysis confirms that refreshing modulated the maintenance of color and shape features in working memory.

**Table 2:** Evidence (BF_10_) for the Effect of Refreshing Status, Cue Type, and their Interaction on Proportion of Correct Recalls of Each Feature in Experiment 3.


RECALLED FEATURE	REFRESHING STATUS	CUE TYPE	TWO-WAY INTERACTION

Location	0.18 (±0.69%)	2.0 × 10^8^ (±0.66%)	0.07 (±0.87%)

Color	98.53 (±0.81%)	2.39 × 10^15^ (±0.78%)	0.04 (±1.57%)

Shape	31.13 (±0.46%)	4.76 × 10^5^ (±0.45%)	0.03 (±0.85%)


We moved next to the analysis of the number of features correctly recalled in each trial. We reasoned that if refreshing modulated the strength of bindings in working memory, then it would increase the probability of recalling both features of the cued object. Figure 7B shows the proportion of trials in which only one (NCorrect = 1) and both features were reported correctly (NCorrect = 2). Refreshing increased the proportion of trials in which both features were recalled correctly (BF_10_ = 2.9 × 10^7^, ±1.0%). Although there was a credible impact of recall cue type (i.e., location, shape, or color; BF_10_ = 8.21 × 10^21^, ±0.91), recall cue type and refreshing status did not interact (BF_10_ = 0.004, ±1.0%). Critically, refreshing had no impact on the number of trials in which a single feature was recalled correctly (BF_10_ = 0.05, ±0.7%). This is in line with the assumption that refreshing served to strengthen feature bindings in working memory. We also compared the refreshing levels aggregated over all cue types: 0-refreshed (*M* = 0.283, *CI* = 0.009) had a lower probability of having both of their features recalled correctly than 1-refreshed (*M* = 0.30, *CI* = 0.007), BF_10_ = 55.4, and 2-refreshed objects (*M* = 0.32, *CI* = 0.01), BF_10_ = 17342. Objects refreshed twice were also more likely to have both of their features recalled correctly than objects refreshed once, BF_10_ = 15.4.

### Discussion

In line with the assumption that attending to a representation in working memory can protect or strengthen feature bindings, we observed that guiding the refreshing of multi-feature objects in working memory increased the proportion of trials in which both features of a multi-feature object were recalled correctly. This provides evidence for the role of attention in the maintenance of feature bindings in working memory. It is worth noting that the results of Experiment 3 are inconsistent with refreshing affecting only individual features. If this was the case, we should have observed that refreshing was particularly effective in improving the recall of location – a feature that was always refreshed when participants used the cues. In contrast to this prediction, location did not show an effect of refreshing, but rather the recall of color and shape (and combinations thereof).

## General Discussion

The aim of the present study was to assess the impact of focusing attention on the representation of multi-feature objects in working memory. We wanted to distinguish two opposing views: attending to representations strengthens the bindings between the features of the object vs. attention strengthens each feature individually. To test these hypotheses, we designed three experiments. In Experiments 1 and 2, we guided the refreshing of multi-feature objects for a recognition test. We observed that refreshing improved the recognition of the intact bindings (match probes), but contrary to our expectations, responses to intrusion probes were insensitive to refreshing frequency. Our initial expectation was that intrusion probes would be either easier or harder to reject depending on whether the bindings or the features were strengthened by attention. Yet, intrusion costs were non-informative regarding the status of the bindings and individual features. One limitation of this study was that intrusion probes always involved the recombination of one presented feature (either shape or color) with a new feature not shown in the current trial. Hence, the lack of familiarity with the not-presented feature (the new feature) might have masked the status of the familiar feature. Hence, in Experiment 3, we employed a dual-feature report task to directly probe for the retrieval of the bindings between the features of the objects, and we did not restrict the bindings to only color and shape, but also included location as an extrinsic feature (Ecker et al., 2013; Goldenhaus-Manning et al., 2024). Experiment 3 showed evidence that attending to objects during the retention interval increased the accessibility of the integrated object: participants were more likely to recall correctly the combination of features of the same object, the more this representation was refreshed.

### Attending to Bindings or Features?

In the present study, we used “think-of” cues to guide the refreshing of multi-feature objects in working memory. We extended our refreshing paradigm to a recognition task and a dual-report task using colored shapes as memoranda. Using these two paradigms, we were able to replicate the refreshing frequency effect previously reported in continuous reproduction tasks (Atkinson et al., 2022; Loaiza & Souza, 2022; Souza et al., 2015, 2018; Souza & Oberauer, 2017; Vergauwe et al., 2023). These results further bolster the utility of using cues to guide attentional refreshing, allowing one to distinguish the effects of varying the amount of attentional deployment to information in working memory.

Here, we asked whether attending to these representations strengthened individual features or feature bindings. We contend that the evidence we obtained is more in line with refreshing strengthening (or protecting) the representation of the integrated, bound object instead of strengthening individual features in working memory. In Experiments 1 and 2, refreshing improved the recognition of match probes which contained the intact binding, whereas the luring effect of individual features remained unaffected by refreshing. This is inconsistent with attention simply acting on individual features. In Experiment 3, refreshing increased the recall of the integrated object, but not of the individual features, again corroborating the view that attention contributes to the maintenance of the integrated representation.

It is worth noting that our findings are inconsistent with the view that reducing attentional resources turns the bindings more fragile, leading to the severance of the integrated representation into their constituent features. Reducing the attentional resources devoted to an object was more likely to reduce the accessibility of the whole object in memory: participants could not recognize the match probes or recall all the features of the probed object (see also Gajewski & Brockmole, 2006).

In sum, directing attention to a representation in working memory increased the accessibility of the bound object, and not simply of its constituent parts. On the other side of the coin, reducing the amount of refreshing did not break the representation into its constituent features. The severance of the bindings (or their fragility) has been considered the currency on which the theories about the role of attention for feature binding have built their case. Our findings seem to lie in the middle. We observed here that attentional refreshing increases the accessibility of the entire object – consistent with it acting on an integrated representation instead of individual features.

### Contradictory Findings on the Role of Attention for Feature Bindings

As reviewed in the introduction, the dual-task paradigm has provided some contradictory findings on the role of attention in feature binding. More recently, a consensus has been emerging regarding the condition in which dual-task costs are more likely to be specifically detrimental to feature bindings. Recent studies showed that the secondary task hinders feature bindings more than individual features when the task taps on object-based attention, for example, by requiring multiple-object tracking or with an object feature report task as in the Duncan task (Elsley & Parmentier, 2009; Fougnie & Marois, 2009; Gao et al., 2017; He et al., 2020; Lu et al., 2019; Shen et al., 2015). In contrast, when the secondary task taps on central attention resources (e.g., counting backwards; tone discrimination) or requires visuospatial attention (e.g., visual search task), dual-task costs tend to be of similar magnitude for the retention of bindings and individual features (Allen et al., 2006, 2012; Goldenhaus-Manning et al., 2024; J. S. Johnson et al., 2008; C. C. Morey & Bieler, 2013; Vergauwe et al., 2014). These results underscore the importance of considering the domain of the attention task, and how disrupting this type of attention contributes to the maintenance of integrated objects.

Our refreshing procedure used spatial cues to guide refreshing, and we were able to guide the sequential refreshing of several objects without leading to the dismantling of the feature bindings of not-cued items (i.e., 0-refreshed items did not increase in the report of single features or were more susceptible to intrusion costs). This might have been the case because our cues demand the deployment of spatial attention without the disruption of object-based attention.

So, does attention contribute to the maintenance of bindings? Yes and no, depending on how one looks at this question. Attention allows one to keep representations in working memory. If the unit is an integrated object, then this is what is strengthened by attention, and not the individual features. If the question is whether attention is necessary for keeping the bindings, and without it, only individual features are retained, then the answer is no. Bindings are not dismantled when attention is withdrawn; rather, the whole object might be lost. We should note that here, we guided spatial attention to promote the refreshing of the representations, and that bindings might be more fragile to disruptions that require object-based attention (or that impose interference at this level).

## Data Accessibility Statement

Materials, Data, and Analysis Scripts are available at: https://osf.io/u9368/.
